# Hypertension in pregnancy and in midlife and the risk of dementia: prospective study of 1.3 million UK women

**DOI:** 10.1002/alz.70595

**Published:** 2025-09-08

**Authors:** Sarah Floud, Carol Hermon, William Whiteley, Kathryn E. Fitzpatrick, Gillian K. Reeves

**Affiliations:** ^1^ Cancer Epidemiology Unit, Nuffield Department of Population Health University of Oxford Oxford UK; ^2^ Centre for Clinical Brain Sciences University of Edinburgh Edinburgh UK; ^3^ Nuffield Department of Population Health University of Oxford, Big Data Institute Oxford UK; ^4^ National Perinatal Epidemiology Unit, Nuffield Department of Population Health University of Oxford Oxford UK

**Keywords:** dementia, hypertension, hypertension in pregnancy, prospective, women

## Abstract

**INTRODUCTION:**

Midlife hypertension is associated with dementia risk, although uncertainties remain regarding its association with subtypes and regarding the effect of pregnancy‐related hypertension on dementia risk.

**METHODS:**

In the Million Women Study, 1,363,457 women (mean age 57) were asked about current treatment for hypertension and hypertension in pregnancy and were followed for first hospital record with any mention of dementia. Cox regression yielded hazard ratios (HRs) adjusted for socioeconomic, lifestyle, and metabolic factors.

**RESULTS:**

With 84,729 dementia cases over 21 years, midlife hypertension was positively associated with dementia (HR 1.17, 95% confidence interval [CI] 1.15 to 1.19); higher for vascular dementia (VaD) (HR 1.50; 95% CI 1.45 to 1.56) than Alzheimer's disease (AD) (HR 1.01; 95% CI 0.98 to 1.04). Hypertension in pregnancy but not in midlife was only weakly associated with dementia (HR 1.04; 95% CI 1.01 to 1.06).

**DISCUSSION:**

Midlife hypertension is a strong risk factor for dementia, largely through VaD. Hypertension during pregnancy does not appear to materially affect dementia risk.

**Highlights:**

- Midlife hypertension was associated with long‐term all‐cause dementia risk.
- Midlife hypertension was associated with VaD, not AD.
- Hypertension in pregnancy has little effect on dementia risk.

## 1 BACKGROUND

Hypertension in midlife (45 to 65 years) is associated with a higher risk of dementia in late life,^1^ but the size of the association varies markedly between cohorts (hazard ratios [HRs] from 1.19 to 2.80).^2^, ^3^, ^4^, ^5^, ^6^, ^7^, ^8^, ^9^, ^10^, ^11^ This variation may be due to differences in length of follow‐up and number of cases. Differential loss to follow‐up of dementia cases could also contribute to this variation.^4^, ^6^, ^7^, ^9^, ^10^ Genetically determined blood pressure (BP) has been associated with dementia risk in some studies,^12^ but not others.^13^ Randomized trials of BP‐lowering treatments to prevent dementia show small or neutral effects.^14^, ^15^ Record‐linkage studies have shown an association between hypertension and vascular dementia (VaD) in particular,^16^, ^17^, ^18^ but population‐based cohort studies, with more extensive control for confounding, have generally been underpowered to reliably assess hypertension‐associated VaD risks.^2^, ^5^, ^6^, ^10^, ^19^ The evidence for Alzheimer's disease (AD) is even more uncertain.^5^, ^17^, ^18^, ^19^, ^20^, ^21^

Hypertension in pregnancy has been reported to be associated with a higher risk of dementia, but it is not clear if this association is causal or due to a poor underlying vascular risk profile that also increases the likelihood of hypertension in midlife and subsequent dementia.^22^ There are few large studies with long‐term follow‐up.^23^, ^24^

The Million Women Study is a prospective study of 1.3 million women aged 50 to 64 years at recruitment, with self‐reported history of hypertension in pregnancy and self‐reported current treatment for hypertension in midlife and an average follow‐up of 21 years. We aimed to estimate the strength of the association between midlife hypertension and all‐cause dementia and its main subtypes (AD, VaD, unspecified dementia), taking into account possible confounding and reverse causation, and to examine whether hypertension during pregnancy confers any additional risk.

## 2 METHODS

### 2.1 Study design and participants

The Million Women Study is a population‐based prospective study.^25^ In median year 1998 (interquartile range [IQR] 1998 to 1999), the study recruited 1.3 million women through the National Health Service (NHS) Breast Screening Programme. Women aged 50 to 64 years were eligible for screening and were sent a postal recruitment questionnaire with their invitation to routine screening, enquiring about sociodemographic, anthropometric, lifestyle, and health factors. About half of the eligible women in the participating screening centers were recruited, which equates to about one in four UK women born in 1935 to 1950 participating in the study. The study population included a wide range of socioeconomic backgrounds and lifestyles broadly typical of UK women of that generation. Resurvey questionnaires were sent to participants every 3 to 5 years. Information on the questionnaires and data access is available on the study website.^26^ Ethical approval was provided by the East of England‐Cambridge South Research Ethics Committee (REC 97/5/001). All participants gave consent for follow‐up through medical records. This study addresses the topic of diversity, equity, and inclusion by focusing on women, who are often underrepresented in research studies.

### 2.2 Procedures and outcomes

Self‐reported treatment for hypertension was first asked about at recruitment in median year 1998 (IQR 1998 to 1999), the baseline for the main analysis. Participants were asked: “Are you now being treated for high blood pressure (hypertension)?” with a yes/no response. Participants were also asked, “Have you ever had high blood pressure in pregnancy?” with a yes/no response. About 3 years later, in median year 2001, participants were asked, “Are you now being treated for high blood pressure” and asked to tick a box if yes and to report the age when the condition was first treated. This enabled us to investigate the duration of hypertension as an additional exposure.

**RESEARCH IN CONTEXT**

1. **Systematic review**: A MEDLINE search identified prospective studies that reported an association between midlife hypertension and all‐cause dementia, but the size of the association varied considerably, as did length of follow‐up. Evidence was also inconsistent for the association with AD and VaD. A recent meta‐analysis reported that hypertension in pregnancy was positively associated with dementia.
2. **Interpretation**: After adjusting for many potential confounders, hypertension in midlife was associated with an increased risk of all‐cause dementia, VaD, and unspecified dementia, but not AD. There was little evidence to suggest bias due to reverse causation. Hypertension during pregnancy had little effect on dementia risk once midlife hypertension was accounted for.
3. **Future directions**: The available evidence suggests that the prevention of hypertension in midlife could lead to a considerable reduction in the incidence of VaD. Interventions should be designed to target prevention of high blood pressure in middle age.

To investigate the validity of self‐reported hypertension, we compared reporting at recruitment with reporting 3 years later in median year 2001. We also compared self‐reports to BP measurements in a resurvey of the cohort about 9 years after baseline, in which 3996 women had their BP measured at local general practitioner (GP) surgeries. Additionally, we compared self‐reports at recruitment to prior records of hypertension in GP records in 72,160 women, using data from a subsample of the cohort that was linked to primary care data (Clinical Practice Research Datalink [CPRD]).

Women were followed‐up for hospital admissions (as day cases or inpatients), deaths, and emigrations through linkage to routinely collected NHS data provided by NHS England (in which up to 20 diagnoses are coded for every hospital admission) and by Public Health Scotland (in which up to six diagnoses are coded for every hospital admission). Hospital diagnoses were coded according to the World Health Organization International Classification of Diseases (ICD)‐10. The main outcome was first mention of any type of dementia (ICD‐10 codes F00‐03 or G30) in a hospital record, with secondary outcomes AD (F00 or G30), VaD (F01), or unspecified (F03), including all four‐character subcategories. There were far fewer cases of the other specific types of dementia. We did not include death records of dementia as an outcome of dementia in our main analysis because of the unknown additional time lag between the diagnosis of dementia and death, but included them in a sensitivity analysis.

### 2.3 Statistical analysis

We used Cox regression models, with time in study as the underlying time variable and stratified by year of birth, year of recruitment, and region to estimate HRs and 95% confidence intervals (CIs) for dementia detected in a hospital record by self‐reported current treatment for hypertension. The baseline for most analyses was the recruitment questionnaire. Women were excluded if they had a mention of dementia in their hospital records before/at recruitment or if they had missing information on self‐reported treatment for hypertension. Person‐years were calculated from baseline to the first of any mention of dementia, emigration or other loss to follow‐up, or end of follow‐up on December 31, 2021.

The models were adjusted, in the first instance, for socioeconomic factors reported at recruitment, including area deprivation (quintiles of Townsend index) and educational qualifications (tertiary, secondary, technical, none but completed compulsory schooling, none and did not complete schooling). Additional adjustment was then made for lifestyle factors reported at recruitment: smoking (never, past, current < 10, current 10 to 19, current 20+ cigarettes/day, not current), alcohol units per week (0, 1 to 2, 3 to 6, 7 to 14, >14 drinks per week), use of menopausal hormone therapy (MHT) (never, past, current), and strenuous exercise (rarely/never, <1, 1 to 3, >3 times per week). Further adjustment was made for body mass index (BMI) (< 20, 20 to 24.9, 25 to 29.9, ≥ 30 kg/m^2^), and self‐reported current treatment for diabetes and high cholesterol at recruitment. For each adjustment variable, missing values were assigned a separate category (≤5% missing for each variable). A sensitivity analysis was conducted that additionally included in the main outcome first events, which were deaths with any mention of dementia in the record.

To investigate the potential impact of reverse causation bias on the association, we split the follow‐up into two periods (<10 and 10+ years). This cut point was chosen based on prior evidence from studies that used neurological examinations to ascertain dementia and found that blood pressure started to fall about 5 years prior to a dementia diagnosis^27^ and on evidence from the Million Women Study that suggested there was on average about 4 years between a diagnosis in primary care and a hospital record of dementia.^28^

Heterogeneity in the associations between hypertension and dementia risk was investigated by estimating the HRs in groups of women cross‐classified by: hypertension and BMI (<25, 25+kg/m^2^), self‐reported treatment for high cholesterol, self‐reported treatment for diabetes, and family history of AD reported at the 2001 questionnaire. Interactions between hypertension and each of these factors were formally assessed using likelihood ratio tests.

The baseline for the analysis of duration of hypertension (no treatment, >0 to 4, 5 to 9, and 10+ years) and dementia risk was the 2001 questionnaire. Adjustments were as described earlier, except treatment for diabetes and high cholesterol were taken from the 2001 questionnaire. A test for trend was conducted within those who received treatment, using mean duration within each category (1.77 years in the >0 to 4 category, 6.61 years in the 5 to 9 category, and 17.40 years in the 10+ category). We excluded women who had reported being treated for hypertension at recruitment but then did not report on hypertension or give an age at first treatment on the 2001 questionnaire.

For the analysis investigating hypertension in pregnancy and risk of dementia, women were excluded if they had a hospital record of dementia before/at recruitment, were nulliparous, or had missing information on parity or hypertension in pregnancy. Using the same adjustments as for the main analysis, HRs for dementia were calculated in relation to history of hypertension in pregnancy and in relation to exposure to both hypertension in pregnancy and in midlife.

Where the results of analyses involving more than two categories are presented (as in the figures), group‐specific CIs are shown to allow direct comparison of risks between categories.^29^ Where two categories of exposure are compared directly (as in the text and in supplementary tables), conventional CIs are given.

To quantify how much of the association between hypertension and VaD was mediated through stroke or transient ischemic attack (TIA), we initially conducted an analysis with additional adjustment for incident stroke/TIA ascertained from self‐reports and hospital admission data during follow‐up as a time‐dependent variable and used the difference method to assess mediation. A more formal mediation analysis was also conducted using a nested case‐control design and the four‐way effect decomposition method,^30^ which allows for a potential interaction between hypertension and stroke/TIA.

To assess the potential for bias in the ascertainment of dementia based on hospital diagnosis, we estimated the cumulative probability of having a hospital record of dementia after a primary care diagnosis in those with and without hypertension using the CPRD sample. We used z‐scores to test for differences in the probability of having a hospital diagnosis within 4 years of a primary care diagnosis by hypertension status. To assess any impact of not detecting all dementia cases in hospital records, we randomly allocated 10% and 20% of the cases to be non‐cases and examined if the associations were changed in the full cohort.

All analyses were performed using StataNow version 18.5 (StataCorp, College Station, TX, USA).

## 3 RESULTS

After excluding women with a record of dementia at recruitment (*n* = 39) and those missing information on hypertension (*n* = 760), there were 1,363,457 women included, with a mean age of 56.7 (SD 4.9) years.

A total of 219,319 (16%) women reported current treatment for hypertension at recruitment. This measure was reasonably stable. When treatment for hypertension at recruitment was compared to that reported 3 years later in median year 2001, 91% of those who reported treatment for hypertension at recruitment still reported being treated for hypertension. In a subsample of the cohort (*n* = 3996) who had their BP measured about 9 years after recruitment, measured systolic and diastolic BP was higher in women who reported current treatment for hypertension at recruitment than in those who did not (systolic BP 140 mmHg treated vs 134 mmHg not treated; diastolic BP 80 vs 78 mmHg, Table A1). In the subsample for whom we had GP records (*n* = 72,160), 90% of women who reported current treatment for hypertension at recruitment, and 15% of women who did not report current treatment for hypertension at recruitment, had a prior record of diagnosis of, or treatment for, hypertension in their GP records. Taken together, these findings suggest that self‐reported treatment for hypertension is a reasonable proxy for hypertension status in this cohort.

Table 1 (and Table A2) shows that compared with women who were not being treated for hypertension, women who were being treated were slightly older, of lower socioeconomic status, less likely to exercise, and more likely to have diabetes and high cholesterol. They were also less likely to smoke or consume alcohol. The largest difference was the rate of obesity (≥30 kg/m^2^), with 32% of those treated for hypertension being obese compared to 15% of those not being treated.

**TABLE 1:** Characteristics of 1,363,457 participants at recruitment by self‐reported current treatment for hypertension and details of follow‐up.

	Self‐reported current treatment for hypertension	
Characteristics at recruitment^a^	Yes	No
No. women (%)	219,319 (16.1)	1,144,138 (83.9)
Mean age (SD)	58.3 (5.0)	56.4 (4.8)
Most deprived fifth (%)	51,886 (23.8)	217,583 (19.2)
No educational qualifications (%)	106,791 (50.2)	474,698 (42.6)
Current smoker (%)	33,594 (15.4)	229,069 (20.2)
≥7 alcoholic drinks per week (%)	43,412 (20.0)	274,173 (24.1)
Current use of MHT (%)	70,330 (32.5)	374,626 (33.1)
Strenuous exercise ≥1 times per week (%)	67,571 (32.2)	441,092 (40.0)
Body mass index ≥30 kg/m^2^ (%)	66,031 (31.9)	164,942 (15.2)
Current treatment for diabetes (%)	15,578 (7.1)	18,144 (1.6)
Current treatment for high cholesterol (%)	25,859 (11.8)	27,933 (2.4)
**Follow‐up**		
Mean years of follow‐up per woman	19.9	21.1
No. dementia cases over full follow‐up	19,273	65,456

^a^ Percentages calculated among those with complete data for each variable. The following variables had small proportions of missing data: deprivation (0.7%), education (2.7%), smoking (0.8%), alcohol (0.8%), use of menopausal hormone therapy (MHT) (1.2%), strenuous exercise (3.7%), body mass index (5.2%).

During a mean follow‐up per woman of 20.9 (SD 4.8) years, there were 84,729 women with a first record of dementia. The maximum period of follow‐up was 26.0 years, during which 283,320 (21%) died and only 1.6% were lost to follow‐up, mainly due to emigration.

Given that BP has been shown to fall in the preclinical phase of dementia,^27^ we first examined the association between hypertension and dementia with the follow‐up split into two periods, but since we did not find a significant difference between the two follow‐up periods (*p* = 0.20), all subsequent analyses used the full follow‐up period (Table A3).

**FIGURE 1:**
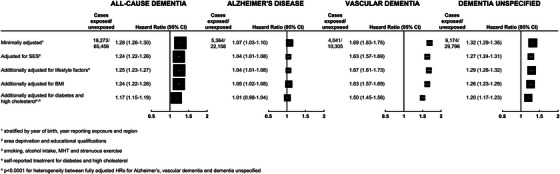
Associations of self‐reported treatment for hypertension with first hospital record of all‐cause dementia, and its subtypes (*N* = 1,363,457).

Figure 1 shows the association between treatment for hypertension reported at recruitment and risk of all‐cause dementia and the three dementia subtypes. Women with hypertension, compared to those without, had a higher incidence of all‐cause dementia (fully adjusted HR 1.17, 95% CI: 1.15 to 1.19). Adjustment for socioeconomic status, lifestyle factors, and BMI slightly attenuated the HR, while there was greater attenuation after adjustment for diabetes and high cholesterol. The results were essentially unchanged in a sensitivity analysis that additionally included the small proportion of cases where dementia was recorded on the death certificate but not in any hospital record (3.8%; *n* = 3314; Table A4).

The fully adjusted HR for hypertension, compared with no hypertension, was highest for VaD (1.50, 95% CI: 1.45 to 1.56), with a weaker association with unspecified dementia (1.20, 95% CI: 1.17 to 1.23). There was no evidence of an association with AD in the fully adjusted model (1.01, 95% CI: 0.98 to 1.04); *p* < 0.0001 for heterogeneity between the fully adjusted HRs for the three subtypes.

**FIGURE 2:**
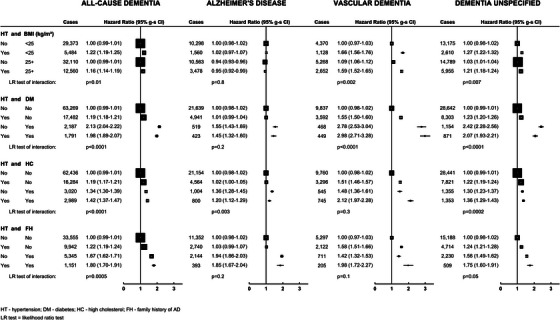
Joint associations of self‐reported treatment for hypertension and body mass index, diabetes, high cholesterol, and family history of AD with first hospital record of all‐cause dementia, and its subtypes.

Interactions between hypertension and BMI, diabetes, high cholesterol, and family history of AD on the risk of all‐cause dementia and its subtypes are shown in Figure 2 (and Table A5). The most notable was the interaction between hypertension and diabetes in relation to risk of dementia in that the positive association of hypertension with dementia risk appears to be largely confined to those without existing diabetes (*p* for interaction between hypertension and diabetes < 0.0001 for all types except AD).

**FIGURE 3:**
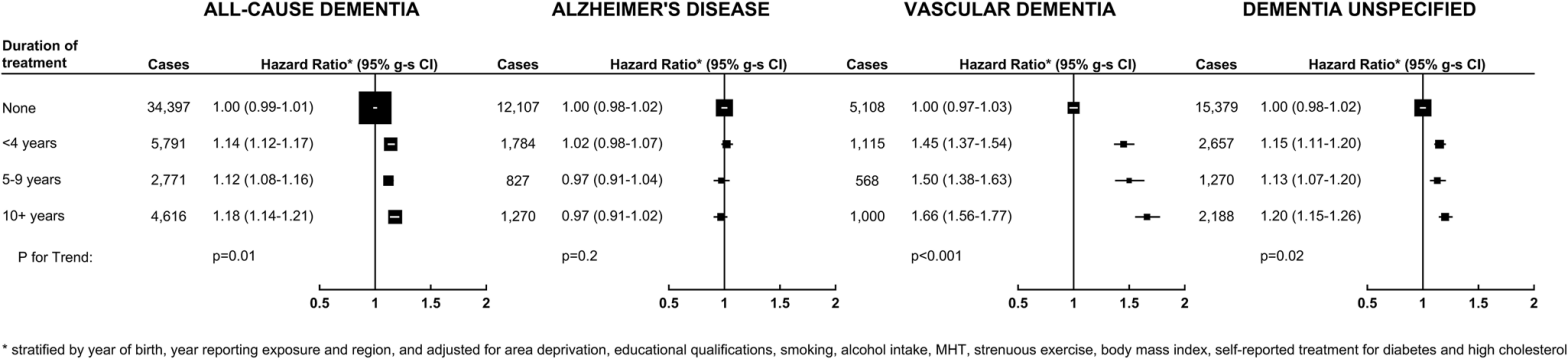
Associations of duration of hypertension treatment with first hospital record of all‐cause dementia, and its subtypes (*N* = 833,012).

Figure 3 (and Table A6) shows the association between duration of hypertension treatment and all‐cause dementia, and its subtypes, in 833,012 women who completed the 2001 questionnaire. There was a significant trend in the association between duration of hypertension and VaD (*p* < 0.001). Compared to those who did not report treatment for hypertension, those who were on treatment for more than 10 years had a HR of 1.66 (95% CI: 1.55 to 1.79) for VaD. The HRs for all‐cause and unspecified dementia were also higher with longer duration of hypertension, whereas there was no association between duration of hypertension and AD.

**FIGURE 4:**
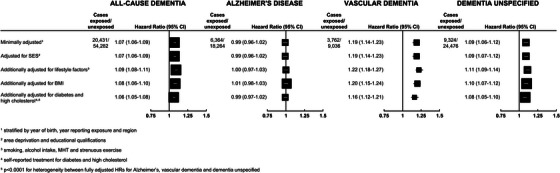
Associations of self‐reported history of hypertension in pregnancy with first hospital record of all‐cause dementia, and its subtypes, in parous women (*N* = 1,210,181).

The association between hypertension during pregnancy and all‐cause dementia and dementia subtypes was also investigated. After excluding nulliparous women (*n* = 146,744) and women with missing information on parity or hypertension during pregnancy (*n* = 6532), there were 1,210,181 women in the analysis, of whom 27% (324,428/1,210,181) reported hypertension during pregnancy. A history of hypertension during pregnancy was associated with higher HRs (fully adjusted) for all‐cause dementia (1.06, 95% CI: 1.05 to 1.08), VaD (1.16, 95% CI: 1.12 to 1.21), and unspecified dementia (1.08, 95% CI: 1.05 to 1.10), but not AD (0.99, 95% CI: 0.97 to 1.02); *p* < 0.0001 for heterogeneity between the fully adjusted HRs for the three subtypes (Figure 4).

**FIGURE 5:**
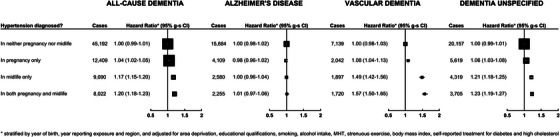
Joint associations of self‐reported history of hypertension in pregnancy and treatment for hypertension at baseline with first hospital record of all‐cause dementia, and its subtypes, in parous women (*N* = 1,210,181).

Since a large proportion of women (27%) who reported a history of hypertension in pregnancy, but only 11% of those without hypertension in pregnancy, also reported being treated for hypertension at baseline, we compared the HRs in women categorized in subgroups defined by both exposures (Figure 5 and Table A7). Compared with women who had no history of hypertension in pregnancy or in midlife, women who reported a history of hypertension during pregnancy but no hypertension in midlife had only a slightly higher HR for all‐cause dementia (1.04, 95% CI: 1.01 to 1.06). In contrast, women with a history of hypertension in pregnancy and midlife had a much higher HR for all‐cause dementia (1.20, 95% CI: 1.17 to 1.23), and this was of similar magnitude to that seen for women who had hypertension in midlife but not in pregnancy (1.17, 95% CI: 1.14 to 1.20). The same pattern of association by exposure subgroup was seen for VaD and unspecified dementia, whereas there was no association with any of the exposure subgroups for AD.

During follow‐up, 132,989 women had a stroke or TIA. The HRs for the association between self‐reported treatment for hypertension and VaD were reduced when adjustment was made for stroke/TIA during follow‐up: the adjusted HR was 1.34 (95% CI: 1.29 to 1.39), suggesting that approximately 28% of the excess risk of VaD related to hypertension was mediated through stroke/TIA. Since there was a modest but significant interaction between hypertension and stroke/TIA (*p* = 0.0039), we also conducted a four‐way decomposition of the effect of hypertension on VaD with stroke/TIA as mediator. This approach yielded a broadly similar estimate of 32% (95% CI: 26 to 37) for the proportion of effect due to mediation.

In a subsample of the cohort with both primary care and hospital records, a total of 948 participants had a first record of dementia in a period with valid and reliable information available from both primary care and hospital records, after excluding 363 whose first hospital record for dementia was before the primary care record. The probability of being admitted to hospital with mention of dementia 4 years after a primary care diagnosis was not found to vary significantly by treatment for hypertension at baseline (cumulative probabilities for those with hypertension 0.60 [95% CI: 0.53 to 0.67] and without hypertension 0.55 [95% CI: 0.52 to 0.59]; *p* = 0.21). In addition, when we randomly allocated cases to be non‐cases in the full cohort, the association between self‐reported treatment for hypertension and all‐cause dementia did not change (10% random allocation: HR 1.18 [95% CI: 1.16 to 1.20]; 20% random allocation: HR 1.17 [95% CI: 1.15 to 1.19]).

## 4 DISCUSSION

This prospective analysis of 1.3 million women supports the hypothesis that hypertension in midlife is associated with an increased risk of all‐cause dementia, even after adjustment for other cardiovascular risk factors such as smoking, BMI, diabetes, and high cholesterol. We have shown that hypertension in midlife was associated with an increased risk of VaD and unspecified dementia, but not AD. The excess risk of VaD was found to be mediated partly through stroke and TIA. The association with VaD was also stronger in those participants who had a longer duration of treatment for hypertension, which is supportive of a causal association. Hypertension during pregnancy was associated with an increased risk of all‐cause dementia, VaD, and unspecified dementia; however, this appeared to be largely due to the association of hypertension during pregnancy with midlife hypertension, since hypertension during pregnancy had little effect on dementia risk once midlife hypertension was considered.

Although a number of studies have reported a statistically significant increased risk of all‐cause dementia in relation to midlife hypertension,^2^, ^3^, ^4^, ^5^, ^6^, ^7^, ^8^, ^9^, ^10^, ^11^ the HR has ranged from 1.19 to 2.80. The Lancet Commission's report on dementia^1^ used a HR of 1.2 (95% CI: 1.1 to 1.4) to estimate the proportion of dementia cases worldwide attributable to hypertension in midlife, which is similar to our HR of 1.17 (95% CI: 1.15 to 1.19). Given that hypertension is primarily associated with VaD, the risk estimate for all‐cause dementia will likely depend on the relative distribution of dementia subtypes in a given population. Other studies have reported a significant increased risk of all‐cause dementia related to increasing BP in middle age.^19^, ^20^, ^31^

Our results showing hypertension in midlife was associated with an increased risk of VaD are broadly consistent with reports from large record‐linkage studies.^16^, ^17^ For example, studies using the CPRD primary care database in England report around a 20% increased risk of VaD associated with a 10‐mmHg increment of systolic BP^16^, ^17^ and diastolic BP in middle age.^16^ However, these studies only adjusted for a limited set of covariates with substantial missing data. Our results are also broadly consistent with results from smaller cohort studies that adjusted for a wider range of confounders, although their HRs ranged from 1.25 to 10.07.^5^, ^6^, ^10^, ^18^, ^19^ Given that hypertension is a major risk factor for stroke and TIA, it is not surprising that hypertension in midlife has been found to be associated with a higher risk of VaD, since a history of stroke is considered in the diagnosis of VaD. However, our study results and results from other analyses^16^ suggest that the association between hypertension and VaD is not wholly mediated through stroke and TIA. It is possible, however, that some of the effect of hypertension on dementia risk may be mediated by more minor cerebrovascular events that were not ascertained through hospital admission data or self‐reports.

To date, the evidence regarding an association between midlife hypertension and AD has been inconsistent. A 2019 meta‐analysis of seven studies found that midlife systolic hypertension was associated with an increased risk of AD, although no association was found for diastolic hypertension.^21^ Two studies published since the meta‐analysis have also reported positive associations for hypertension with AD,^19^, ^20^ but two other studies reported null associations with AD,^5^, ^17^ in agreement with our results. The evidence so far suggests that a null association between hypertension and AD is a reasonable conclusion, given that many of the studies that did not see an association had, at the same time, sufficient statistical power to detect a significant positive association with VaD,^5^, ^6^, ^10^, ^17^ as we did. It is interesting to note that two studies reported associations with AD that differed by sex, but again there was inconsistency: in one study the positive association was found only in men^18^ and in the other the positive association was found only in women.^19^

Most previous studies did not examine the risk of dementia in relation to duration of exposure to hypertension in midlife, apart from two, both of which reported higher risks in those with longer exposure to hypertension in middle age, consistent with our findings.^8^, ^20^ The Whitehall II study modeled trajectories of exposure to hypertension from age 45 to 61 in 8313 participants and reported a HR of 1.29 (95% CI: 1.00 to 1.66) for dementia for those with longer exposure to hypertension compared to those with low or no exposure to hypertension.^8^ The Korean NHIS‐HealS cohort reported a HR for dementia per 10% increase in duration of hypertension of 1.09 (95% CI: 1.08 to 1.10) for 292,822 participants aged 40 to 59 years at recruitment.^20^

Most previous studies that reported an association between hypertension in pregnancy and dementia did not have measures of midlife hypertension. When adjustment was made for cardiovascular disease in a Swedish register‐based study,^23^ the positive association between pregnancy‐induced hypertension and all‐cause dementia was attenuated and became non‐significant, whereas for VaD the significant association remained, although somewhat attenuated. In a Danish register‐based cohort study,^24^ the strong positive associations between pre‐eclampsia and all‐cause dementia and VaD were also attenuated but remained statistically significant after adjusting for cardiovascular disease, stroke, hypertension, chronic kidney disease, and diabetes developing during follow‐up. However, neither study had sufficient statistical power to compare the risks in subgroups defined by both hypertension in pregnancy and midlife hypertension, which allows for the assessment of the independent effect of hypertension in pregnancy on dementia in those with similar midlife hypertension.

The proportion of participants (16%) who reported treatment for hypertension matched the prevalence of women aged 55 to 64 years treated for hypertension in the 1998 Health Survey for England (HSE) in women,^32^ suggesting that our findings are generalizable to the wider population. A limitation, however, is the lack of direct measures of BP at baseline, which, as well as identifying women who had high BP but were not being treated, would also indicate if the hypertension was successfully controlled by treatment. We have shown that self‐reported treatment for hypertension is likely to be a reliable proxy for diagnosed hypertension, but a key clinical question is the degree to which uncontrolled hypertension, rather than treatment for hypertension, increases the risk of dementia. Given that our exposed group included women whose hypertension was successfully controlled (in the 1998 HSE,^32^ about a third of treated women were normotensive), our estimated associations probably represent a lower bound for the effect of uncontrolled hypertension on dementia risk. We relied upon maternal recall of hypertension during pregnancy, which has low sensitivity but high specificity.^33^ In our cohort, the proportion of participants (27%) who reported hypertension during pregnancy was much higher than the 8% to 10% prevalence of hypertensive disorders of pregnancy expected according to the National Institute for Clinical Excellence (NICE) guideline (NG133), but it is similar to the 25% of pregnant women in the Northern Finland Birth Cohort who had pre‐eclampsia, gestational hypertension, or new‐onset hypertension which did not meet the clinical criteria for hypertension.^34^ It is therefore likely that in the Million Women Study we are seeing the effect of hypertension around the time of pregnancy, regardless of the cause. We were not able to distinguish between subtypes of hypertensive disorder during pregnancy, although the most recent meta‐analysis suggests little difference in risk estimates by subtype.^22^ A further limitation is that the use of hospital records for dementia ascertainment may not capture all cases of dementia, but we included dementia mentioned anywhere in a hospital admission, not only the cause of admission, and we had minimal loss to follow‐up. Furthermore, a validation study in this cohort showed that 95% of diagnoses of dementia could be confirmed in primary care records.^35^ While not all participants with dementia are admitted to hospital, we showed previously that half of those with a primary care diagnosis of dementia had a hospital admission with mention of dementia within 4 years,^36^ and we have shown here that this did not differ by hypertension status. Hence, the large majority of women classified as not having dementia are likely to have been correctly classified, and any misclassification does not appear to be biased by hypertension status. Moreover, our simulation exercise, where we randomly misclassified up to 20% of cases as non‐cases, did not find any change in the associations. Finally, although we adjusted for a range of potential confounders, we cannot rule out residual confounding due to the observational nature of the study.

Hypertension during midlife is a risk factor for all‐cause dementia, particularly VaD, but does not appear to increase the risk of AD. Although hypertension during pregnancy was associated with a small increased risk of all‐cause dementia and VaD, this may be due to a shared poor underlying vascular risk profile with hypertension in midlife, since hypertension during pregnancy had little effect on dementia risk once hypertension in middle‐age was considered. Measures targeted at preventing hypertension in middle age are likely to be most successful at reducing dementia incidence.

## CONFLICT OF INTEREST STATEMENT

The authors declare no competing interests. Author disclosures are available in the supporting information.

## Supporting information



Supporting Information

Supporting Information
