# Supplementary material for: Hypertension in pregnancy and in midlife and the risk of dementia: prospective study of 1.3 million UK women
Source: Alzheimers Dement. 2025 Sep 8;21(9):e70595. doi: 10.1002/alz.70595 (PMC12417322; doi:10.1002/alz.70595)
Supplement: Supplementary file 2 — Supporting Information [file ALZ-21-e70595-s001.docx]

**SUPPLEMENTARY MATERIAL**

**Hypertension in pregnancy and in midlife and the risk of dementia: prospective study of 1.3 million UK women**

Authors: Sarah Floud PhD^1^, Carol Hermon MSc^1^, William Whiteley PhD^2,3^, Kathryn E Fitzpatrick DPhil^4^, Gillian K Reeves PhD^1^

Affiliations:

1. Cancer Epidemiology Unit, Nuffield Department of Population Health, University of Oxford

2. Centre for Clinical Brain Sciences, University of Edinburgh

3. Nuffield Department of Population Health, University of Oxford

4. National Perinatal Epidemiology Unit, Nuffield Department of Population Health, University of Oxford

**Table A1** Self-reported and measured blood pressure by self-reported hypertension at recruitment (using data from the Disease Susceptibility in Women sub-cohort of the Million Women Study)

|  | **Self-reported hypertension requiring treatment at recruitment** | |
| --- | --- | --- |
|  | No | Yes |
| Reported systolic BP in mmHg (n=1975), mean (SD) | 132 (15) | 139 (16) |
| Reported diastolic BP in mmHg (n=1948), mean (SD) | 78 (9) | 81 (9) |
| Measured systolic BP in mmHg (n=3937), mean (SD) | 134 (16) | 140 (16) |
| Measured diastolic BP in mmHg (n=3935), mean (SD) | 78 (10) | 80 (10) |

**Table A2: Characteristics of 1,363,457 participants at recruitment by self-reported current treatment for hypertension and details of follow-up**

|  | **Hypertension** | |
| --- | --- | --- |
| Characteristics at recruitment* | Yes | No |
| No. of women (%) | 219319 (16.1) | 1144138 (83.9) |
| Mean age (SD) | 58.3 (5.0) | 56.4 (4.8) |
| Deprivation quintile, n (%) |  |  |
| Q1, Least deprived | 37761 (17.3) | 234469 (20.6) |
| Q2 | 40219 (18.5) | 231358 (20.4) |
| Q3 | 42213 (19.4) | 227766 (20.1) |
| Q4 | 45676 (21.0) | 224510 (19.8) |
| Q5, Most deprived | 51886 (23.8) | 217583 (19.2) |
| Education attainment†, n (%) |  |  |
| Tertiary | 21314 (10.0) | 154548 (13.9) |
| Secondary | 48242 (22.7) | 298990 (26.8) |
| Technical | 36314 (17.1) | 185969 (16.7) |
| None, completed compulsory schooling | 100237 (47.1) | 448943 (40.3) |
| None, did not complete compulsory schooling | 6554 (3.1) | 25755 (2.3) |
| Smoking, n (%) |  |  |
| Never | 109202 (50.2) | 546373 (48.1) |
| Past | 61934 (28.5) | 303928 (26.8) |
| Current <10 cigarettes/day | 8785 (4.0) | 57827 (5.1) |
| Current 10-19 cigarettes/day | 16351 (7.5) | 114425 (10.1) |
| Current 20+ cigarettes/day | 8458 (3.9) | 56817 (5.0) |
| Not current‡ | 12710 (5.8) | 56326 (5.0) |
| Alcohol, drinks per week, n (%) |  |  |
| 0 | 65421 (30.1) | 260838 (23.0) |
| 1-2 | 68243 (31.4) | 349657 (30.8) |
| 3-6 | 40362 (18.6) | 250704 (22.1) |
| 7-14 | 33819 (15.6) | 216116 (19.0) |
| >14 | 9593 (4.4) | 58057 (5.1) |
| MHT use, n (%) |  |  |
| never | 108797 (50.4) | 564494 (49.9) |
| past | 36946 (17.1) | 191935 (17.0) |
| current | 70330 (32.5) | 374626 (33.1) |
| Strenuous exercise, times/week, n (%) |  |  |
| Rarely/never | 118657 (56.5) | 522680 (47.4) |
| <1 | 23608 (11.3) | 139076 (12.6) |
| 1-3 | 57310 (27.3) | 369318 (33.5) |
| >3 | 10261 (4.9) | 71774 (6.5) |
| BMI, kg/m², n (%) |  |  |
| <20 | 3832 (1.9) | 45295 (4.2) |
| 20- | 57646 (27.9) | 494494 (45.6) |
| 25- | 79355 (38.4) | 380706 (35.1) |
| ≥30 | 66031 (31.9) | 164942 (15.2) |
| Treatment for diabetes at recruitment, n (%) |  |  |
| no | 203741 (92.9) | 1125994 (98.4) |
| yes | 15578 (7.1) | 18144 (1.6) |
| Treatment for high cholesterol at recruitment, n (%) | |  |
| no | 193460 (88.2) | 1116205 (97.6) |
| yes | 25859 (11.8) | 27933 (2.4) |
| **Follow-up** |  |  |
| Mean years of follow-up per woman | 19.9 | 21.1 |
| No. of dementia cases over full follow-up | 19273 | 65456 |

*Percentages calculated among those with complete data for each variable. The following variables had small proportions of missing data: deprivation (0.7%), education (2.7%), smoking (0.8%), alcohol (0.8%), MHT use (1.2%), strenuous exercise (3.7%), body mass index (5.2%).

† See publication for full explanation of the educational qualifications variable: Floud, S., Balkwill, A., Moser, K. et al. The role of health-related behavioural factors in accounting for inequalities in coronary heart disease risk by education and area deprivation: prospective study of 1.2 million UK women. BMC Med 14, 145 (2016). <https://doi.org/10.1186/s12916-016-0687-2>

‡ Not current smoker refers to women who reported that they were not currently smoking but did not say if they were an ex-smoker or never smoker.

**Table A3.** Associations of self-reported treatment for hypertension with first hospital record of all-cause dementia, by period of follow-up (N=1,363,457)

|  | Interval after reporting hypertension (years) | | | | |
| --- | --- | --- | --- | --- | --- |
|  | <10 | | 10+ | | |
| Cases exposed/unexposed | 1075/3328 | | 18198/62128 | | |
| Follow-up years (mean for cases) | 7.20 | | 18.68 | | |
|  |  | |  | | |
| HR (95% CI)* | 1.12 (1.04-1.21) | | 1.18 (1.16-1.20) | | |
|  |  | |  | | |
| Test for heterogeneity | Chi-square=1.62 p=0.20 | | | | |
|  | |  | |  |  |

* stratified by year of birth, year reporting exposure and region, and adjusted for area

deprivation, educational qualifications, smoking, alcohol intake, MHT, strenuous exercise,

body mass index, self-reported treatment for diabetes and high cholesterol

**Table A4.** Associations of self-reported treatment for hypertension with first hospital record of all-cause dementia, including dementia mentioned on death certificates (N=1,363,457)

|  | Cases exposed/unexposed | HR | 95% CI |
| --- | --- | --- | --- |
|  | 20,021/68,022 |  |  |
| Minimally adjusted† |  | 1.28 | (1.26-1.30) |
| Fully adjusted‡ |  | 1.18 | (1.16-1.19) |

† stratified by year of birth, year reporting exposure and region

‡ stratified by year of birth, year reporting exposure and region and adjusted for area deprivation, educational qualifications, smoking, alcohol intake, MHT, strenuous exercise, body mass index, self-reported treatment for diabetes and high cholesterol

**Table A5 Results underlying Figure 2** (Joint associations of self-reported treatment for hypertension and body mass index, diabetes, high cholesterol and family history of AD with first hospital record of all-cause dementia, and its subtypes) **showing conventional and group-specific confidence intervals**.

|  | ALL CAUSE DEMENTIA | | ALZHEIMER’S DISEASE | | VASCULAR DEMENTIA | | DEMENTIA, UNSPECIFIED | |
| --- | --- | --- | --- | --- | --- | --- | --- | --- |
|  | HR (95% CI) | HR (95% g-s CI) | HR (95% CI) | HR (95% g-s CI) | HR (95% CI) | HR (95% g-s CI) | HR (95% CI) | HR (95% g-s CI) |
| **HT and BMI (kg/m²)** |  |  |  |  |  |  |  |  |
| No <25 | 1.00 - | 1.00 (0.99-1.01) | 1.00 - | 1.00 (0.98-1.02) | 1.00 - | 1.00 (0.97-1.03) | 1.00 - | 1.00 (0.98-1.02) |
| Yes <25 | 1.22 (1.18-1.26) | 1.22 (1.19-1.25) | 1.02 (0.96-1.07) | 1.02 (0.97-1.07) | 1.66 (1.55-1.77) | 1.66 (1.56-1.76) | 1.27 (1.21-1.32) | 1.27 (1.22-1.32) |
| No 25+ | 1.00 (0.98-1.02) | 1.00 (0.99-1.01) | 0.94 (0.92-0.97) | 0.94 (0.93-0.96) | 1.09 (1.04-1.13) | 1.09 (1.06-1.12) | 1.03 (1.00-1.05) | 1.03 (1.01-1.04) |
| Yes 25+ | 1.16 (1.14-1.19) | 1.16 (1.14-1.19) | 0.95 (0.92-0.99) | 0.95 (0.92-0.99) | 1.59 (1.51-1.67) | 1.59 (1.52-1.65) | 1.21 (1.17-1.25) | 1.21 (1.18-1.24) |
| **HT and DM** |  |  |  |  |  |  |  |  |
| No No | 1.00 - | 1.00 (0.99-1.01) | 1.00 - | 1.00 (0.98-1.02) | 1.00 - | 1.00 (0.98-1.02) | 1.00 - | 1.00 (0.99-1.01) |
| Yes No | 1.19 (1.17-1.22) | 1.19 (1.18-1.21) | 1.01 (0.98-1.05) | 1.01 (0.99-1.04) | 1.55 (1.49-1.61) | 1.55 (1.50-1.60) | 1.23 (1.20-1.26) | 1.23 (1.20-1.26) |
| No Yes | 2.13 (2.04-2.22) | 2.13 (2.04-2.22) | 1.55 (1.42-1.70) | 1.55 (1.43-1.69) | 2.78 (2.53-3.05) | 2.78 (2.53-3.04) | 2.42 (2,28-2.57) | 2.42 (2.28-2.56) |
| Yes Yes | 1.98 (1.88-2.08) | 1.98 (1.89-2.07) | 1.45 (1.32-1.60) | 1.45 (1.32-1.60) | 2.98 (2.71-3.29) | 2.98 (2.71-3.28) | 2.07 (1.93-2.22) | 2.07 (1.93-2.21) |
| **HT and HC** |  |  |  |  |  |  |  |  |
| No No | 1.00 - | 1.00 (0.99-1.01) | 1.00 - | 1.00 (0.98-1.02) | 1.00 - | 1.00 (0.98-1.02) | 1.00 - | 1.00 (0.99-1.01) |
| Yes No | 1.19 (1.17-1.21) | 1.19 (1.17-1.21) | 1.02 (0.99-1.06) | 1.02 (1.00-1.05) | 1.51 (1.45-1.58) | 1.51 (1.46-1.57) | 1.22 (1.18-1.25) | 1.22 (1.19-1.24) |
| No Yes | 1.34 (1.29-1.39) | 1.34 (1.30-1.39) | 1.36 (1.28-1.45) | 1.36 (1.28-1.45) | 1.48 (1.36-1.62) | 1.48 (1.36-1.61) | 1.30 (1.23-1.37) | 1.30 (1.23-1.37) |
| Yes Yes | 1.42 (1.37-1.47) | 1.42 (1.37-1.47) | 1.20 (1.12-1.29) | 1.20 (1.12-1.29) | 2.12 (1.96-2.29) | 2.12 (1.97-2.28) | 1.36 (1.28-1.44) | 1.36 (1.29-1.43) |
| **HT and FH** |  |  |  |  |  |  |  |  |
| No No | 1.00 - | 1.00 (0.99-1.01) | 1.00 - | 1.00 (0.98-1.02) | 1.00 - | 1.00 (0.97-1.03) | 1.00 - | 1.00 (0.98-1.02) |
| Yes No | 1.22 (1.19-1.24) | 1.22 (1.19-1.24) | 1.03 (0.98-1.07) | 1.03 (0.99-1.07) | 1.58 (1.50-1.67) | 1.58 (1.51-1.66) | 1.24 (1.20-1.29) | 1.24 (1.21-1.28) |
| No Yes | 1.67 (1.62-1.72) | 1.67 (1.62-1.71) | 1.94 (1.86-2.04) | 1.94 (1.86-2.03) | 1.42 (1.31-1.53) | 1.42 (1.32-1.53) | 1.56 (1.49-1.63) | 1.56 (1.49-1.62) |
| Yes Yes | 1.80 (1.70-1.91) | 1.80 (1.70-1.91) | 1.85 (1.67-2.05) | 1.85 (1.67-2.04) | 1.98 (1.72-2.28) | 1.98 (1.72-2.27) | 1.75 (1.60-1.91) | 1.75 (1.60-1.91) |
|  |  |  |  |  |  |  |  |  |

HT - hypertension: DM - diabetes: HC - high cholesterol: FH - family history of AD

LR test - likelihood ratio test

**Table A6 Results underlying Figure 3** (Associations of duration of hypertension treatment with first hospital record of all-cause dementia, and its subtypes (N=833012)) **showing conventional and group-specific confidence intervals**

|  |  |  |  |  |  |  |  |  |
| --- | --- | --- | --- | --- | --- | --- | --- | --- |
|  | ALL CAUSE DEMENTIA | | ALZHEIMER’S DISEASE | | VASCULAR DEMENTIA | | DEMENTIA, UNSPECIFIED | |
| Duration of treatment | HR* (95% CI) | HR* (95% g-s CI) | HR* (95% CI) | HR* (95% CI) | HR* (95% CI) | HR* (95% CI) | HR* (95% CI) | HR* (95% CI) |
| None | 1.00 - | 1.00 (0.99-1.01) | 1.00 - | 1.00 (0.98-1.02) | 1.00 - | 1.00 (0.97-1.03) | 1.00 - | 1.00 (0.98-1.02) |
|  |  |  |  |  |  |  |  |  |
| < 4 years | 1.14 (1.11-1.18) | 1.14 (1.12-1.17) | 1.02 (0.97-1.08) | 1.02 (0.98-1.07) | 1.45 (1.36-1.55) | 1.45 (1.37-1.54) | 1.15 (1.11-1.20) | 1.15 (1.11-1.20) |
|  |  |  |  |  |  |  |  |  |
| 5-9 years | 1.12 (1.08-1.17) | 1.12 (1.08-1.16) | 0.97 (0.91-1.05) | 0.97 (0.91-1.04) | 1.50 (1.37-1.64) | 1.50 (1.38-1.63) | 1.13 (1.07-1.20) | 1.13 (1.07-1.20) |
|  |  |  |  |  |  |  |  |  |
| 10+ years | 1.18 (1.14-1.22) | 1.18 (1.14-1.21) | 0.97 (0.91-1.03) | 0.97 (0.91-1.02) | 1.66 (1.55-1.79) | 1.66 (1.56-1.77) | 1.20 (1.15-1.26) | 1.20 (1.15-1.26) |
|  |  |  |  |  |  |  |  |  |

* stratified by year of birth, year reporting exposure and region, and adjusted for area deprivation, educational qualifications, smoking, alcohol intake,

MHT, strenuous exercise, body mass index, self-reported treatment for diabetes and high cholesterol

**Table A7 Results underlying Figure 5** (Associations of self-reported history of hypertension in pregnancy and treatment for hypertension at baseline with first hospital record of all-cause dementia, and its subtypes, in parous women (N=1210181)) **showing conventional and group-specific confidence intervals**

|  | | ALL CAUSE DEMENTIA | | ALZHEIMER’S DISEASE | | VASCULAR DEMENTIA | | DEMENTIA, UNSPECIFIED | |
| --- | --- | --- | --- | --- | --- | --- | --- | --- | --- |
| Hypertension diagnosed | | HR* (95% CI) | HR* (95% g-s CI) | HR* (95% CI) | HR* (95% g-s CI) | HR* (95% CI) | HR* (95% g-s CI) | HR* (95% CI) | HR* (95% g-s CI) |
| In neither pregnancy nor midlife | | 1.00 - | 1.00 (0.99-1.01) | 1.00 - | 1.00 (0.98-1.02) | 1.00 - | 1.00 (0.98-1.03) | 1.00 - | 1.00 (0.99-1.01) |
|  | |  |  |  |  |  |  |  |  |
| In pregnancy only | | 1.04 (1.01-1.06) | 1.04 (1.02-1.05) | 0.98 (0.95-1.02) | 0.98 (0.96-1.02) | 1.08 (1.03-1.14) | 1.08 (1.04-1.13) | 1.06 (1.03-1.09) | 1.06 (1.03-1.08) |
|  | |  |  |  |  |  |  |  |  |
| In midlife only | | 1.17 (1.14-1.20) | 1.17 (1.15-1.20) | 1.00 (0.96-1.04) | 1.00 (0.96-1.04) | 1.49 (1.42-1.57) | 1.49 (1.42-1.56) | 1.21 (1.17-1.25) | 1.21 (1.18-1.25) |
|  | |  |  |  |  |  |  |  |  |
| In both pregnancy and midlife | | 1.20 (1.17-1.23) | 1.20 (1.18-1.23) | 1.01 (0.97-1.06) | 1.01 (0.97-1.06) | 1.57 (1.49-1.66) | 1.57 (1.50-1.65) | 1.23 (1.18-1.27) | 1.23 (1.19-1.27) |
|  |  |  |  |  |  |  |  |  |  |
